# Only the strong survive: therapeutic selective pressure drives medulloblastoma leptomeningeal metastasis

**DOI:** 10.1002/1878-0261.70125

**Published:** 2025-09-16

**Authors:** Francis Y. He, Adrienne Boire

**Affiliations:** ^1^ Neuroscience Program, Graduate School of Biomedical Science Weill Cornell Medicine New York USA; ^2^ Human Oncology & Pathogenesis Program Memorial Sloan Kettering Cancer Center New York USA; ^3^ Department of Neurology Memorial Sloan Kettering Cancer Center New York USA

**Keywords:** leptomeningeal metastasis, medulloblastoma, radiation therapy

## Abstract

Medulloblastoma (MB) is the most common malignant tumor in the central nervous system in childhood and regularly metastasizes to the leptomeninges following radiation therapy. Using patient‐derived medulloblastoma models and genetically engineered mouse models, Nör *et al.* observed enhanced inflammation and infiltration of myeloid cells within the brain following irradiation. The authors identified inflammatory cytokines and the resulting breakdown of blood–brain barriers as the main culprits of MB leptomeningeal metastasis. This study demonstrated that targeting inflammation through the use of dexamethasone effectively reduced systemic inflammatory cytokines and the resulting radiation‐induced leptomeningeal metastasis.

## Graphical abstract

Radiation therapy induces cancer cell death and immune cell infiltration, mainly monocytes, macrophages, and dendritic cells. Elevated serum cytokine levels and disruption of the blood–brain barrier promote leptomeningeal dissemination. Cotreatment with dexamethasone reduces cytokine levels, preserves barrier integrity, and limits metastatic spread, resulting in decreased overall disease burden.

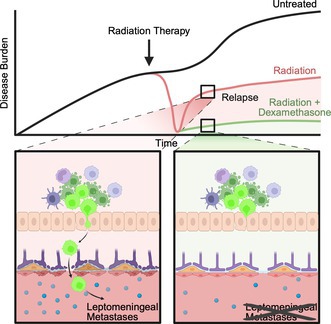

**Abbreviations**
BBB: blood–brain barrier
CNS: central nervous system
CSF: cerebrospinal fluid
CTCs: circulating tumor cells
CyToF: cytometry by time‐of‐flight
LM: leptomeningeal metastasis
LPS: lipopolysaccharide
MB: medulloblastoma
RNA‐seq: RNA sequencing
RT: radiation therapy

Radiation therapy (RT) has long been a cornerstone of medulloblastoma treatment, introduced to prong survival after surgical resection to counteract local recurrence and metastasis to the leptomeninges, (the pia, arachnoid, and cerebrospinal fluid, CSF). However, irradiation of the central nervous system (CNS) carries substantial side effects and remains a major cause of neurocognitive morbidity in medulloblastoma survivors [1]. Recent work from Nör *et al*. investigates the unintended consequences of radiation therapy, mechanistically linking this treatment to subsequent leptomeningeal metastasis (LM) [2]. The provocative work raises a critical question in cancer care: Does radiation therapy for medulloblastoma treatment uphold the principle of “do no harm,” or does the treatment select for more aggressive disease?

Surgical resection and radiation remain first line of treatment for medulloblastoma. The authors first evaluated prognosis across multiple clinical trials combining radiation therapy with cytotoxic chemotherapy. Surprisingly, radiation therapy correlated with a significant increase in LM incidence. This was investigated mechanistically in an experimental series employing patient‐derived xenograft models, establishing the causal relationship between RT and the development of LM. Addressing the next question of how this might occur, the authors established how RT reshapes the tumor microenvironment. Within 5 h post irradiation, the cancer cells undergo apoptosis, and bulk RNA‐seq reveals enrichment in immune‐related pathways, particularly those associated with the innate immune response. Complementary single cell RNA‐seq and Cytometry by Time‐Of‐Flight (CyToF) analyses uncovered enrichment in immune cell subpopulations, particularly myeloid populations including monocytes, macrophages, and dendritic cells.

The team next turned their attention to the endothelial compartment. Radiation was found to disrupt tight junction integrity and increase blood–brain barrier (BBB) permeability. Notably, RT also increased the number of circulating tumor cells (CTCs) in peripheral blood in flank xenograft models and elevated serum inflammatory markers (Fig. 1). Previous work by the group reported the presence of CTCs from medulloblastoma into the periphery. These cells, competent to metastasize back into the leptomeninges, rely on the monocyte attractant cytokine CCL2 [3]. Again, the authors find that myeloid‐derived inflammatory cytokines play an instrumental role in leptomeningeal spread. Postulating that radiation‐provoked inflammation might exacerbate metastasis by inducing inflammation, the authors induced inflammation using lipopolysaccharide (LPS), which remarkably phenocopied the prometastatic effects of RT, promoting LM. Conversely, treatment with dexamethasone, a steroid commonly used in the clinical setting, reduced inflammation and blocked LM (Fig. 1).

**Fig. 1:**
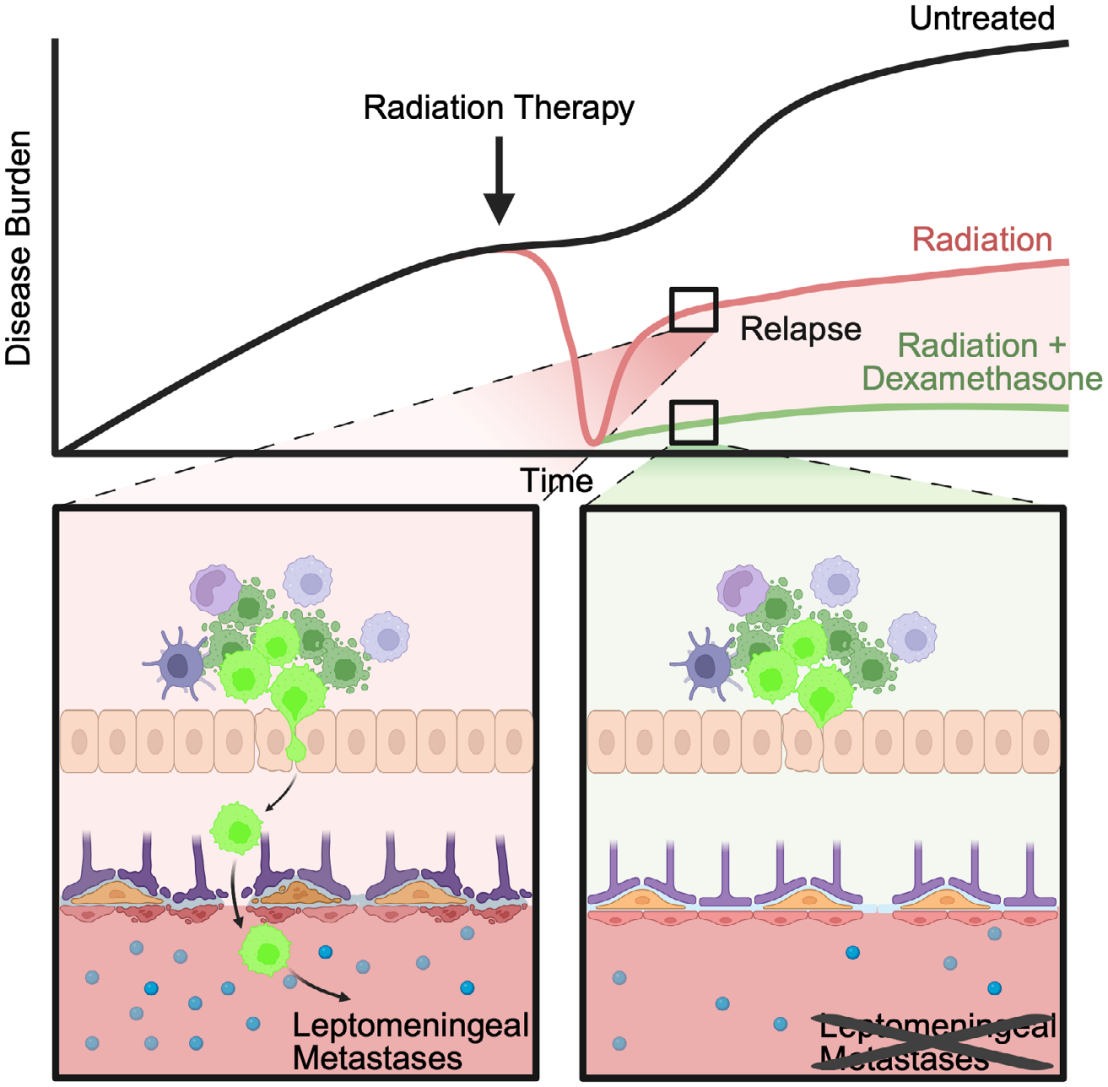
Radiation therapy induces cancer cell death and immune cell infiltration, mainly monocytes, macrophages, and dendritic cells. Elevated serum cytokine levels and disruption of blood–brain barrier promotes leptomeningeal dissemination. Cotreatment with dexamethasone reduces cytokine levels, preserves barrier integrity and limits metastatic spread, resulting in decreased overall disease burden. Created in BioRender. He, F. (2025) https://BioRender.com/qi7mi6n

Medulloblastoma metastasis represents a complex, whole organism process encompassing the tumor‐cell intrinsic features, the local cerebellar microenvironment, the leptomeningeal space, and the systemic circulation. While Nör *et al*. shed light on the inflammatory consequences of RT, important questions remain. Importantly, can we leverage the potential benefit of immune therapy (provoking inflammation in the space) without incurring increased risk of leptomeningeal spread? Addressing this question will require granular characterization of the antitumor response in the space in comparison with the post RT response. In addition, while the authors beautifully demonstrate increased vascular permeability post RT, does this treatment alter the leptomeningeal niche to support medulloblastoma growth? Might RT promote one aspect of the metastatic cascade and inhibit another? Nör *et al*. argue that the prometastatic effect of RT is secondary to the influx of immune cells into the CNS, triggered by tumor cell death and the subsequent clearance of cellular debris, a process that inadvertently heightens inflammation [2]. Might similar mechanisms apply for other solid tumors after RT?

As treatments become more efficacious, and patients live longer, the consequences of the selective pressure of treatment on malignancy become more apparent. This has been observed most readily in the case of tumors treated with targeted therapies: In prostate cancer and EGFR‐mutant lung adenocarcinomas, targeted therapies including antiandrogen treatment and EGFR inhibitors are effective at controlling primary disease [4, 5]. However, these treatments favor the emergence of neuroendocrine phenotypes that were vanishingly rare prior to these therapies [6]. In the context of medulloblastoma, increased mutational burden and pathways such as TP53 have emerged following treatment [7, 8]. Nör *et al*. invite us to consider the possibility that therapies such as RT may exert selective pressure indirectly, through impacting the tumor microenvironment, to select for metastatic cells.

The work also challenges us to consider our use of RT in the treatment of medulloblastoma. RT remains one of the most effective tools for eliminating residual tumor cells and preventing recurrence, particularly in high‐risk, group 3, group 4 patients. The long‐term cost of RT is substantial; neurocognitive impairment, hearing loss, and impaired quality of life are frequently observed among survivors [1]. These adverse effects are common and significantly impact the patient's ability to live an independent life. Thus, proton radiation, RT avoidance, or reduced dose are often preferable for younger patients [9, 10]. Importantly, RT alone does not protect against disease progression, as Nor *et al*. demonstrate; rather, it shifts the pathology from local recurrence to metastatic disease [2, 7].

These findings underscore the need to examine our approach to the use of RT in medulloblastoma as well as other CNS tumors. RT‐mediated alteration of the local microenvironment may well reprogram both the micro‐ and macroenvironment, leading to organism‐wide changes that alter the biology of these malignancies. This work suggests that assessing response to RT through imaging measures alone likely overlooks important alterations in the local and systemic inflammatory response. These changes are doubtless present in the context of other CNS malignancies. It is time to conduct thoughtful, biopsy‐rich clinical trials to capture local and distal environmental changes resulting from RT so that we may anticipate and 1 day avoid metastatic progression.

## Conflict of interest

AB holds an unpaid position on the Scientific Advisory Board for Evren Scientific and is an inventor on the following patents: 62/258044, 10/413522, and 63/052139. FH declares no conflict of interest.

## Author contribution

FH and AB conceived and wrote the manuscript.
